# Draft genome sequence data of *Vibrio harveyi* VH1 isolated from a diseased tiger grouper, *Epinephelus fuscoguttatus*, cultured in Malaysia

**DOI:** 10.1016/j.dib.2022.108533

**Published:** 2022-08-08

**Authors:** Md. Ali Amatul-Samahah, Aslah Mohamad, Nurhidayu Al-saari, Mohd Zamri-Saad, Mohamad Noor Amal Azmai, Mohd Termizi Yusof, Md. Yasin Ina-Salwany, Mami Tanaka, Sayaka Mino, Tomoo Sawabe

**Affiliations:** a Department of Aquaculture, Faculty of Agriculture, Universiti Putra Malaysia, 43400 Serdang, Selangor, Malaysia; b Freshwater Fisheries Research Division, Fisheries Research Institute Glami Lemi, Jelebu 71650, Malaysia; c Aquatic Animal Health and Therapeutics Laboratory, Institute of Bioscience, Universiti Putra Malaysia, 43400 Serdang, Selangor, Malaysia; d International Institute for Halal Research And Training, International Islamic University Malaysia (IIUM), 53100 Jalan Gombak, Selangor, Malaysia; e Department of Veterinary Laboratory Diagnosis, Faculty of Veterinary Medicine, Universiti Putra Malaysia, 43400 UPM Serdang, Selangor, Malaysia; f Department of Biology, Faculty of Science, Universiti Putra Malaysia, 43400 UPM Serdang, Selangor, Malaysia; g Department of Microbiology, Faculty of Biotechnology and Biomolecular Sciences, Universiti Putra Malaysia, 43400 UPM Serdang, Selangor, Malaysia; h Laboratory of Microbiology, Faculty of Fisheries Sciences, Hokkaido University, 3-1-1 Minato-cho, Hakodate 041-8611, Japan

**Keywords:** Aquaculture, Vibriosis, Grouper, Genome, *Vibrio sp.*

## Abstract

Vibriosis accounts for 66.7% of diseases reported in groupers' cultures and affects almost all stages of growth. The disease could lead up to mortality up to 50% mortality, and it was reported that high stocking density and poor fish handling were among the factors that contributed to the disease dissemination. *V. harveyi* has been reported to be among the causative agent and has caused acute mortality in cage groupers. In this study, we report the genome of *V. harveyi* VH1 isolated from a diseased tiger grouper *Epinephelus fuscoguttatus*, reared in a cage farm located in the coastal area of Langkawi.

**Specification Table**

**Table:** 

Subject	Biology
Specific subject area	Microbiology, Genomics, Biotechnology
Type of data	Table, figures
How the data were acquired	The draft genome sequence was processed using MinION instrument, Oxford Nanopore Technology, UK.
Data format	Raw, analyzed and deposited
Description of data collection	*Vibrio harveyi* VH1 was isolated from the skin lesion samples originated from a cultured tiger grouper (*Epinephelus fuscoguttatus*) in Malaysia. Genomic DNA extraction and sequencing were performed.
Data source location	*Vibrio harveyi* VH1 was isolated from the skin lesion samples originated from a cultured tiger grouper (*Epinephelus fuscoguttatus*) in a cage farm located in the coastal area of Langkawi, Kedah, Malaysia (latitude and longitude: 6°13′16.1″N 99°46′07.5″E). *Vibrio harveyi* VH1 genome was analyzed at the Aquatic Animal Health and Therapeutics Laboratory, Institute of Bioscience, Universiti Putra Malaysia, 43400 Serdang, Selangor, Malaysia (latitude and longitude: 2° 59′ 59.1684″ N, 101° 43′ 21.918″ E).
Data accessibility	Data are publicly available at NCBI GenBank https://www.ncbi.nlm.nih.gov/nuccore/JAAIXX000000000 https://www.ncbi.nlm.nih.gov/biosample/SAMN14091020 https://www.ncbi.nlm.nih.gov/bioproject/PRJNA606422
Related research article	Y.K. Chin, M.Y. Ina-Salwany, M. Zamri-Saad, M.N.A. Amal, A. Mohamad, J.Y. Lee, S. Annas & N. Al-saari, Efficacy of bath vaccination with a live attenuated *Vibrio harveyi* against vibriosis in Asian seabass fingerling, *Lates calcarifer,* Disease of Aquatic Organism 137: 167-173 (2020). https://doi.org/10.3354/dao03435

## Value of the Data

• The draft genome of *Vibrio harveyi* VH1, isolated from cultured marine fish (*E. fuscoguttatus*) will be useful for further research on the virulence gene transfer of *V. harveyi* and *Vibrio* spp. in general.
• Data on the genome sequence of *Vibrio harveyi* VH1 can be used for comparative genomic studies with other *Vibrio* spp. disease isolates from other places.
• Data on the genome sequence of *Vibrio harveyi* VH1 could be used to identify and characterize important virulence factors that contribute to the pathogenesis.
• Data is useful for the bioinformatician and bacteriologist to better understand the genetic features of *Vibrio harveyi* VH1 and novel insights about its key virulence determinants.

## 1 Data Description

**Fig. 1:**
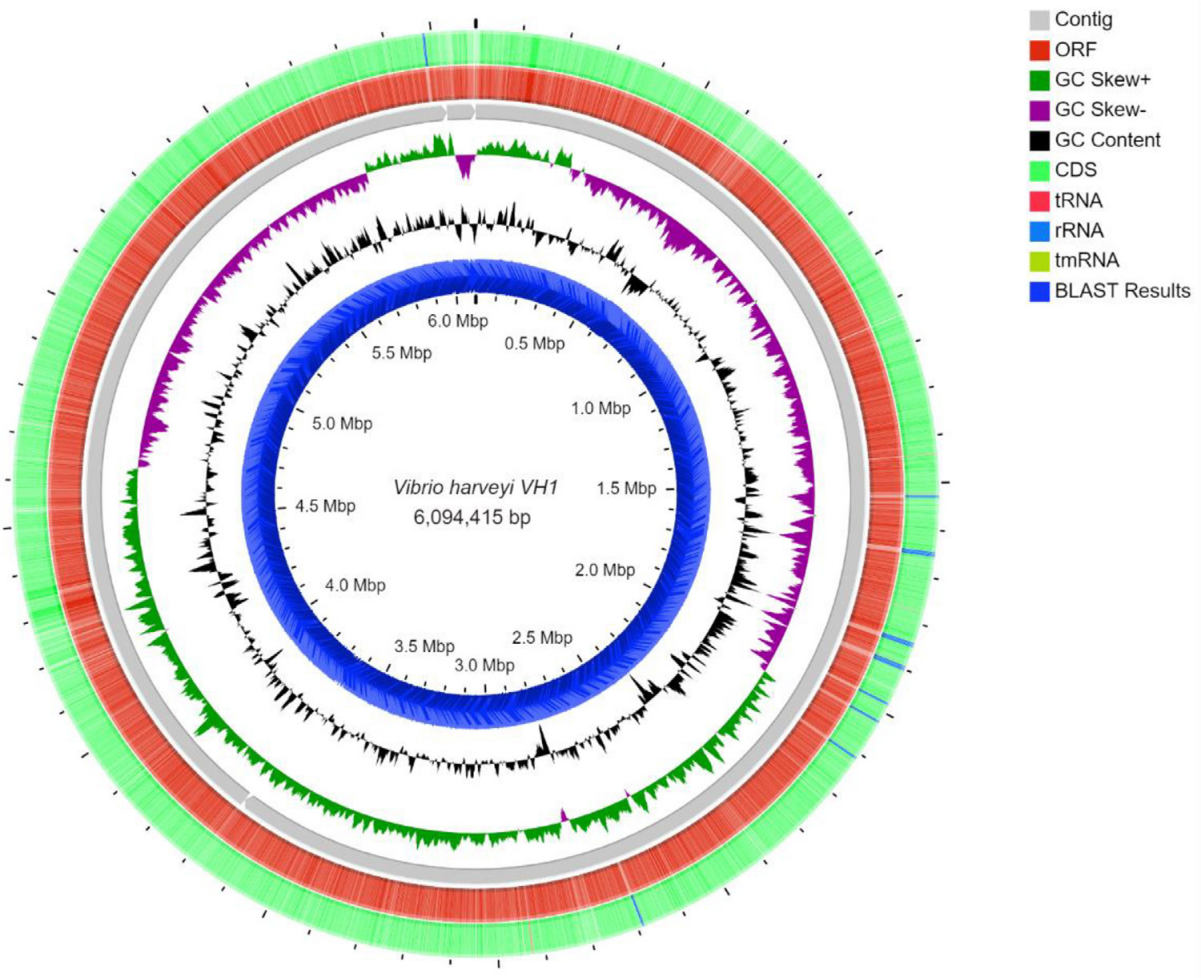
Circular map of the *V. harveyi* VH1 genome. From the outermost circle to the center: CDSs on forward strand (including tRNA, rRNA and mRNA), open reading frame (ORF), Contigs, GC skew+ and GC skew-, GC content, BLAST results, and the marker of genome size is the innermost circle.

**Fig. 2:**
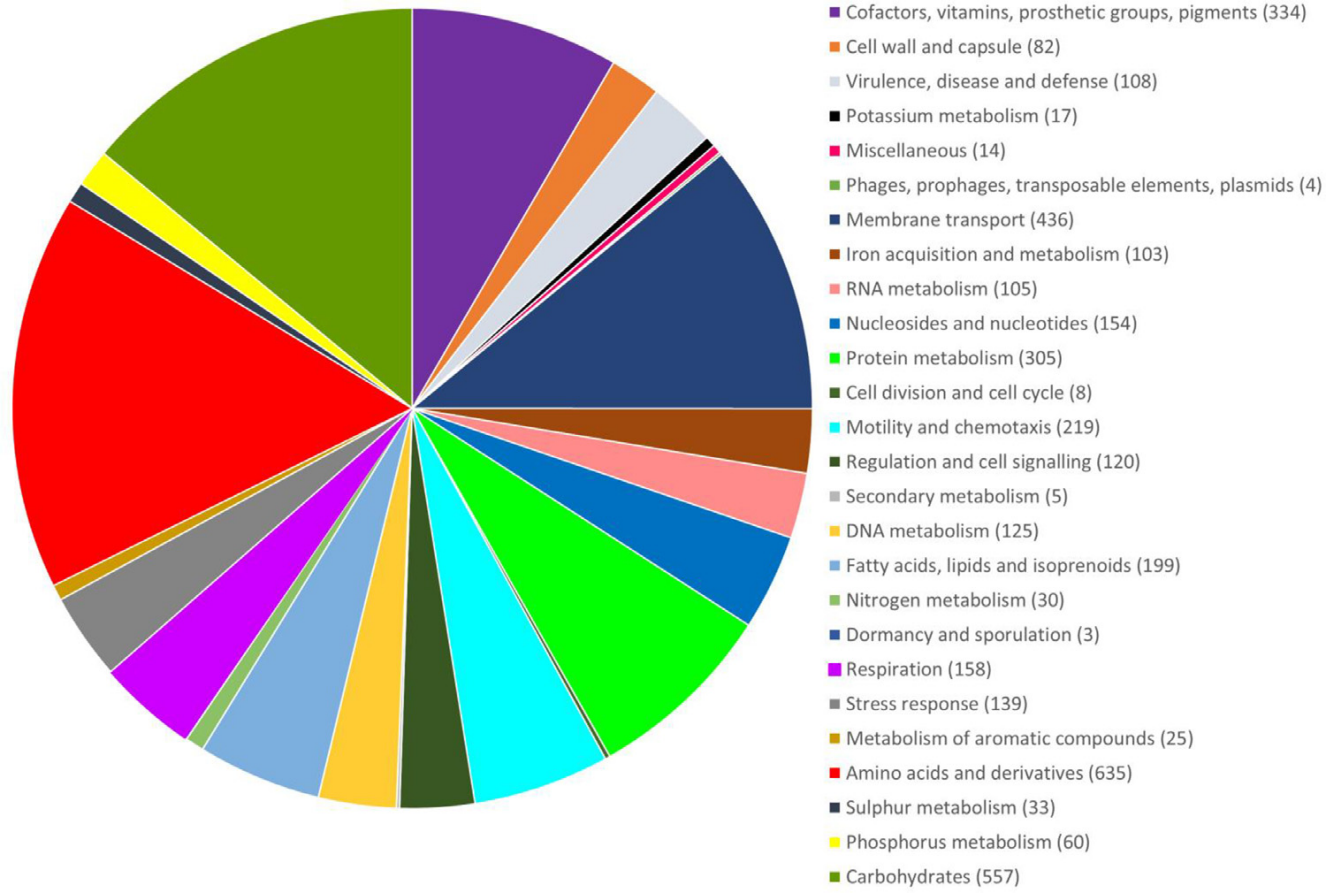
The subsystem category distribution of *V. harveyi* VH1 based on the SEED viewer RAST.

The draft genome of *V. harveyi* strain VH1 was reported in this finding. The draft genome assembly of *V. harveyi* strain VH1 has a length of 6,094,415 bp and a GC content of 44.8% (Fig. 1). The paired-end reads were assembled *de novo* into three huge contigs using Canu 1.6 with error and mismatch correction (N50, 3,675,737 bp). Annotation of the draft genome with RAST (Rapid Annotation using Subsystem Technology) 2.0 identified 416 subsystems, 8763 coding sequences (CDS), and 161 total RNAs in the genome (Table 2). 108 coding sequences involves in virulency, diseases, and defense which inclusive of bacteriocins productions, ribosomally synthesized antibacterial peptides, and resistance to antibiotics (Fig. 2). Four coding sequences were found to have identity with phages, prophages, transposable elements and plasmids [1], [2], [3], [4].

**Table 1:** General information and genome sequencing project information of *V. harveyi* VH1.

Items	Description	
Classification	Domain	Bacteria
	Phylum	Proteobacteria
	Class	Gammaproteobacteria
	Order	Vibrionales
	Family	Vibrionaceae
	Genus	Vibrio
	Species	*Vibrio harveyi*
Gram stain	Negative	
Cell shape	Rod-shaped	
Pigmentation	Non-pigmented	
Sporulation	Non-sporulating	
Optimum temperature	25 °C	
Salinity	30 ppt	
Oxygen	Aerobic	
MIGS Data		
Submitted to NCBI	GenBank	
Investigation type	Bacteria	
Project Name	Genome of *Vibrio harveyi* VH1	
Collection date	May 2017	
Longitude and Latitude	6°13′16.1"N 99°46′07.5"E	
Geographic location name	Langkawi, Kedah, Malaysia	
Environment biome	Coastal area	
Environment feature	Cage farm	
Environment material	Water	
Depth	3.0-5.0 m	
Biotic relationship	Free living

**Table 2:** Genome features of *V. harveyi* VH1.

Attribute	Description
Genome size (bp)	6,094,415 bp
G+C content (%)	44.8%
CDS (coding sequences)	8763
rRNA number	37
tRNA number	124
Genbank accession	JAAIKJ000000000
BioSample acession	SAMN14091020
BioProject acession	PRJNA606422

Besides that, 219 coding sequences involved in motility and chemotaxis, 120 coding sequences involved in regulation and cell signaling, 139 coding sequences involved in stress response, and 158 coding sequences involved in cell respiration. The genome sequence of *V. harveyi* VH1 serves as an additional genomic resource for comparative genomic studies of other *V. harveyi* strains that infected marine fish (Fig. 2).

## 2 Experimental Design, Materials and Methods

*V. harveyi* strain VH1 was isolated from the skin lesion samples originated from a male diseased tiger grouper *Epinephelus fuscoguttatus*, reared in a cage farm located in the coastal area of Langkawi, Malaysia (Table 1). The *V. harveyi* isolate was cultured and maintained in thiosulfate-citrate-bile salts (TCBS) (Oxoid) agar and tryptone soy broth (TSB) (Oxoid), supplemented with NaCl (1.5% w/v) at 30 °C. A TCBS agar is a selective medium for enteropathogenic *Vibrio* spp. When cultured on TCBS agar, the colonies of pathogenic *V. harveyi* strain VH1 appeared as yellow colonies. Genomic DNA of *V. harveyi* VH1 was extracted from the culture using the DNA kit (Thermo Fisher Scientific). Sequencing library was prepared using the Rapid Barcoding Kit (SQK-RBK001) (Oxford Nanopore Technologies, Oxford, UK) as per instruction in the manual provided by the manufacturer. The library was then loaded to a MinION R9 flow cell (FLO-MIN106) (Oxford Nanopore Technologies, Oxford, UK), and the sequencing analysis was performed using MinKNOW software version 1.7.14. Fast5s from Nanopore sequencing were basecalled with ONT Albacore Sequencing Pipeline software version 2.0.2 and reads passing the internal test were used for subsequent analysis [5]. Porechop 0.2.2 (https://github.com/rrwick/Porechop) was used for debarcoding and adaptor trimming. Nanopore reads were assembled using Canu 1.6 [6]. For Nanopore-only assembly, output contigs were polished using Nanopolish software version 0.8.1 (https://github.com/jts/nanopolish). Contigs from Canu 1.6 were manually closed based on the assembly graph with Bandage software version 0.8.1 [7].

## Ethics Statements

The study was conducted according to the guidelines by the Animal Care and Use Committee Universiti Putra Malaysia (UPM/IACUC/AUP-R078/2019). All animal experiments are reported in compliance with the ARRIVE guidelines and carried out in accordance with the U.K. Animals (Scientific Procedures) Act, 1986 and associated guidelines, EU Directive 2010/63/EU for animal experiments, or the National Institutes of Health guide for the care and use of Laboratory animals (NIH Publications No. 8023, revised 1978).

## CRediT Author Statement

The authors here declare their individual contributions:

**Md. Ali Amatul-Samahah:** Writing – original draft and editing; **Aslah Mohamad:** Investigation and writing; **Nurhidayu Al-saari:** Investigation and writing; **Mohd Zamri-Saad:** Validation and reviewing; **Mohamad Noor Amal Azmai:** Validation and reviewing; **Mohd Termizi Yusof:** Validation and reviewing; **Ina-Salwany Md.Yasin:** Validation, reviewing, editing & supervision; **Mami Tanaka:** Investigation, data curation, software & validation; **Sayaka Mino:** Data curation, software, validation & investigation; **Tomoo Sawabe:** Software, validation, investigation & supervision.

## Declaration of Competing Interest

The authors declare that they have no known competing financial interests or personal relationships that could have appeared to influence the work reported in this paper.

## Data Availability

Draft genome sequence data of Vibrio harveyi VH1 (Original data) (NCBI).
